# Impact of Fermented Corn–Soybean Meal on Gene Expression of Immunity in the Blood, Level of Secretory Immunoglobulin A, and Mucosa-Associated Bacterial Community in the Intestine of Grower–Finisher Pigs

**DOI:** 10.3389/fvets.2020.00246

**Published:** 2020-06-02

**Authors:** Junfeng Lu, Mengqing Zhu, Haigang Cao, Xuan Zhang, Zhaolu Wang, Xiaoyu Zhang, Xiao Li, Jianhong Hu, Gongshe Yang, Xin'e Shi

**Affiliations:** Key Laboratory of Animal Genetics, Breeding and Reproduction of Shaanxi Province, Laboratory of Animal Fat Deposition and Muscle Development, College of Animal Science and Technology, Northwest A&F University, Yangling, China

**Keywords:** fermented feed, gut health, immunity, mucosa-associated microbiota, pig

## Abstract

This study was conducted to determine the effect of a fermented corn–soybean meal [fermented feed (FF)] on the gene expression of immunity in the blood, the level of secretory immunoglobulin A (sIgA), and mucosa-associated bacterial community in the duodenum and colon of grower-finisher pigs. In this study, crossbred barrows (Duroc × Landrace × Large White) were randomly assigned to either an unfermented corn–soybean diet (Ctrl) (*n* = 6) or an FF diet (*n* = 6), and then the following were examined: the expression of immunity using real-time reverse transcription polymerase-chain reaction in the blood, sIgA using enzyme-linked immunosorbent assay (ELISA), and changes in the bacterial community using Illumina Hiseq sequencing in the mucosa of the duodenum and colon. Compared with control pigs fed with a standard diet, the results showed that FF caused upregulation of the mRNA expression of Toll-like receptor 3 (TLR3), TLR4, TLR6, and TLR8 in the blood (*P* < 0.05). Moreover, sequencing of 16S rRNA genes in duodenal mucosa samples indicated that the FF diet had a lower proportion of Tenericutes (*P* < 0.05) in the duodenal mucosa-associated microbiota, and FF significantly increased the percentage of Rikenellaceae and Christensenellaceae but decreased the abundance of Lachnospiraceae (*P* < 0.05) in the colonic mucosa-associated microbiota. The ELISA results showed that FF significantly increased the concentration of sIgA in the colonic mucosa (*P* < 0.05). More importantly, our correlation analysis indicated that the gene expression of immunity in the blood and the concentration of sIgA was associated with colonic mucosa-associated microbiota. Our data provide new knowledge into the adaptation response of the intestine to fermented feeding in monogastric animals.

## Introduction

Fermentation has been used to prepare healthy human foods for a long time (1, 2). In the livestock industry, fermented feed (FF) has generated special attention because it is associated with the improvement of nutritional quality (3), with the reduction of antinutritional factors (4). More importantly, FF also provides probiotics, beneficial microbiota, and metabolites for animals (5, 6). Furthermore, fermentation could increase amino acid and phosphorus digestibility in soybean meal (7). In recent years, research on finding alternatives to antibiotics in feed and reducing feed costs has continually promoted the development of FF for probiotics (8). Using FF could improve the growth performance of nursery (9) and weaned piglets (10). Meanwhile, FF has also been observed to improve the growth performance of their progeny and decrease diarrhea incidence (11). FF is beneficial to swine nutrition and has been successfully applied in several European countries such as Denmark. Also adding luster to its growing profile is the recent report that FF has beneficial effects on modulating pig gut microbiota (12, 13).

The gut microbiota of pigs strongly affects gastrointestinal and systemic health (14, 15). Using next-generation sequencing, extensive studies have been conducted to characterize the gut microbiota, primarily using fecal samples. However, little work has been done on the more proximal regions of the gut or on the microbiota living in the outer mucosal layer, which may differ from the fecal microbiome. Mucosa-associated microbiota, residing within the outer mucus layer, plays an important role in the intestine (16, 17). Researchers found that mucosa-associated microbiota is vital in exploring bacterial-triggered host immunity and gut–brain communication (18). In addition, although the effects of diet/dietary supplements on gut microbiota have become apparent, little research has been carried out on the effect of diet on mucosa-associated microbiota. Therefore, it is important to understand the effect of diet supplements on mucosa-associated microbiota (small intestine and large intestine) and serum immunity.

This study was part of a larger project investigating the effect of an FF meal on the whole-body health of pigs. The main study tested the hypothesis that a fermented complete commercial soybean diet will regulate gut health. Therefore, in this study, we mainly focused on the effect of FF on the mucosa-associated microbiota and gene expression of immunity in the blood. We hypothesized that feeding FF to pigs could affect the gene expression of immunity in the blood, the level of secretory immunoglobulin A (sIgA), and the mucosa-associated microbiota in the intestine of grower–finisher pigs. The potential links between colonic mucosa-associated microbiota modulation and gene expression of immunity and concentration of sIgA of FF intake are also discussed herein. These results are important for a better understanding of the composition of mucosa-associated microbiota in the intestine and how FF influences the mucosal health of the entire gastrointestinal tract of monogastric animals.

## Materials and Methods

### Ethics Statement

Animal protocols for this study were approved by the Animal Ethics Committee of Northwest A&F University (Yangling, Shaanxi, China).

### Diets and Animals

**Figure 1:**
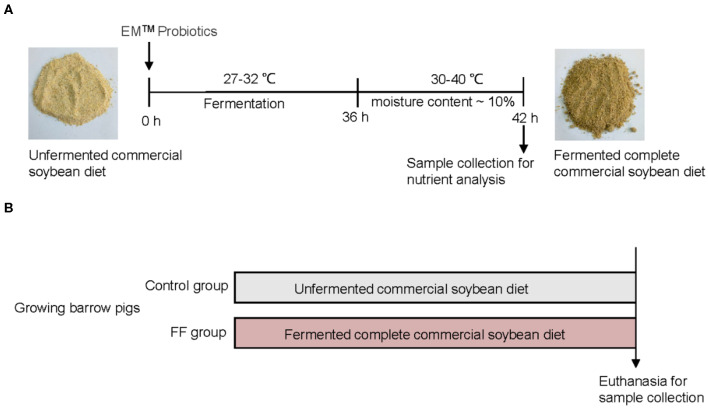
Experimental design. **(A)** Preparation and composition of FF. **(B)** Experimental design.

The preparation of FF was performed as previously described (19) and shown in Figure 1A. Briefly, the corn–soybean meals (Beijing Dabeinong, Beijing, China) were fermented with probiotics (Nongfukang, Zhengzhou, China) at 27–32°C for 36 h according to the manufacturer's instructions. After fermentation, the FF meal was dried until the moisture content was nearly 10%. The ingredients and nutrient composition of experimental meals are shown in Supplementary Table 1.

A total of 48 growing barrow pigs [(Duroc × (Landrace × Large White)] were allocated randomly to two groups fed with either a corn–soybean diet or a fermented complete commercial soybean diet (Figure 1B). Pigs were housed in a controlled environment (four pigs per pen, six pens per group) where water and feed were provided *ad-libitum*. There were no significant differences in terms of body weights between the two groups (53.19 ± 2.17 vs. 54.60 ± 1.62 kg) at the beginning of the feeding trial. All pigs remained healthy and showed no signs of enteric or metabolic disturbances.

### Sample Collection

Upon reaching slaughter weight (~110 kg), one pig from each pen was randomly selected and fasted overnight (sampling number of pigs were six in each group). For sample collection, 1-ml whole blood samples were collected from the external jugular vein of pigs and then immediately mixed with 3-ml RNA Lock Reagent (Bioteke Co., Beijing, China). The mixture was kept at room temperature for 2 h and then stored at −80°C until use. Then, serum samples were carefully collected and centrifuged at 2,500 rpm for 15 min at 4°C; afterward, the samples were brought back to the laboratory for further analysis. Meanwhile, the pigs were anesthetized and slaughtered. Mucosal scrapings from the duodenum and colon were removed, quickly frozen, and then stored at −80°C.

### Real-Time Quantitative Reverse Transcription-Polymerase Chain Reaction

Total RNA extraction from blood was performed using RNA Lock Reagent (Tiangen Biotech, Beijing, China) according to the manufacturer's instructions. Reverse transcription (RT) and quantitative RT polymerase chain reaction (qRT-PCR) were performed as previously described (20). All measurements were performed in triplicate. The qRT-PCR primers are shown in Supplementary Table 2.

### Enzyme-Linked Immunosorbent Assay (ELISA) Analysis of Interleukin-10, Tumor Necrosis Factor-α, and sIgA

For serum interleukin-10 (IL-10) and tumor necrosis factor-alpha (TNF-α) measurements, serum was measured using a swine IL-10 and TNF-α ELISA kit (Abcam, Cambridge, UK) as per the manufacturer's protocols.

For duodenal mucosa and colonic mucosa sIgA measurements, the loop of the colon was obtained and gently rinsed with 0.9% saline; then, the digest was carefully removed to obtain the mucosa samples and immediately placed in 1.5-ml centrifuge tubes. The concentration of sIgA was measured using a swine IgA ELISA kit (Abcam) following the manufacturer's instructions. Each sample was tested in triplicate.

### 16S rRNA High-Throughput Sequencing of Mucosa-Associated Microbiota

Bacterial genomic DNA (gDNA) was extracted from the duodenal mucosa and colonic mucosa samples using the bacterial gDNA stool mini-kit (Qiagen, Hilden, Germany), and the quality of the DNA was verified with visual monitoring after 1% agarose gel electrophoresis. According to the V4–V5 hypervariable region of the 16S rRNA genes, universal primers with barcode (Supplementary Table 3) were used to amplify the DNA sequences, followed by generation of sequencing libraries using the TruSeq DNA PCR-free sample preparation kit. Next, the libraries were sequenced on an Illumina Hiseq platform (performed by Novogene Biological Information Technology Co., Beijing, China). Raw data were submitted to the National Center of Biotechnology Information Sequence Read Archive Database (https://www.ncbi.nlm.nih.gov/sra) with accession no. PRJNA607631.

The paired-end reads were merged using Fast Length Adjustment of Short (FLASH) reads software. After quality filtering using QIIME 1.7.02, effective reads were finally obtained by removing the chimera sequences using the UCHIME algorithm. The sequences were then aligned into the UPARSE software (Uparse v7.0.1001) of bacterial 16S rRNA genes, and the operational taxonomic units (OTUs) were identified as one cluster at the 97% similarity level. Next, MUSCLE software (Version 3.8.31) was used to obtain an abundance-based coverage estimator (ACE) and the Shannon index. The different relative abundances of bacterial taxa within different groups [false discovery rate (FDR) <0.05] from the inner to outer rings with phylum and family were measured.

### Statistical Analysis

Statistical analyses were performed using SPSS software (v.20.0; IBM Corp., Armonk NY, USA), and values are presented as means ± standard error of the mean (SEM). The data of genes related to immunity, inflammatory factors, and sIgA concentrations were evaluated using Student's *t*-test. The data of duodenal mucosa and colonic mucosa microbiota were evaluated using non-parametric Kruskal–Wallis test followed by *post-hoc* test, and the *P*-values were corrected using FDR as previously described (21). FDR values <0.05 were considered significantly different. The correlations between colonic mucosa microbial composition and genes related to immunity in blood and concentration of sIgA in the colonic mucosa, which was significantly influenced by FF, were evaluated using Spearman's correlation test. The Pearson correlation coefficient was >0.5, and *P* < 0.05 were considered to be significantly different.

## Results

### Effect of FF on Expression of Genes Related to Immunity in the Blood and Serum Inflammatory Factor

**Figure 2:**
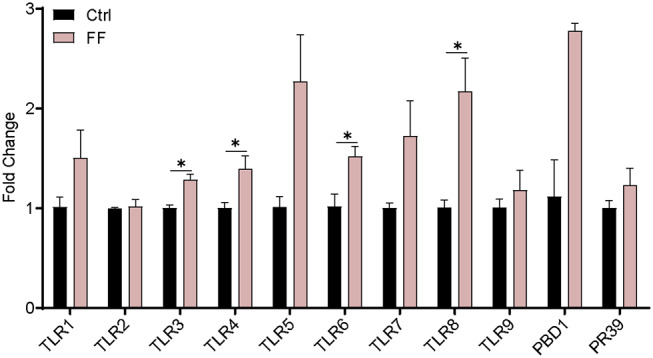
Effect of FF on the expression of genes related to blood immunity. The Ctrl group was designed as 1-fold change. Ctrl, pigs fed with normal commercial feed; FF, pigs fed with fermented meal. Data are presented as mean ± SEM (*n* = 6). ^*^P < 0.05.

**Figure 3:**
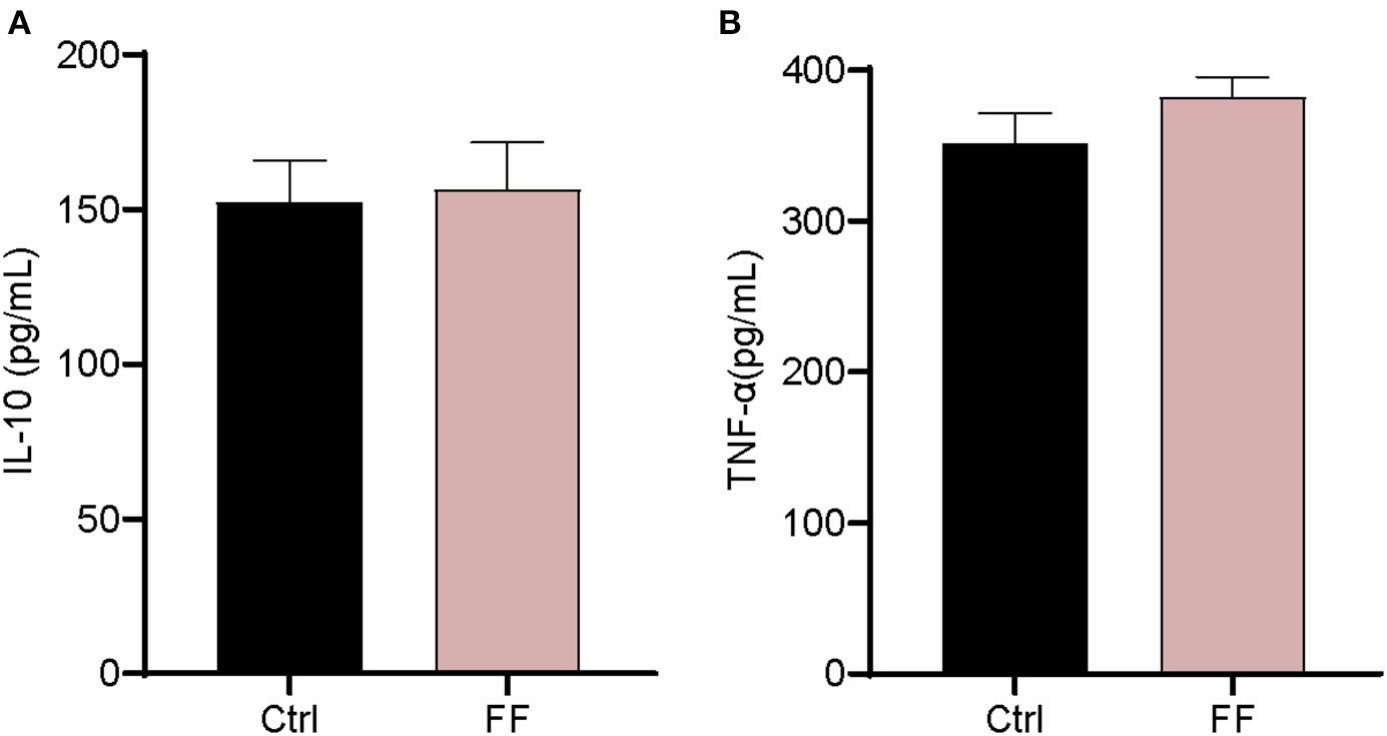
Effect of FF on the serum cytokine levels. **(A)** IL-10; **(B)** TNF-α. Ctrl: pigs fed with normal commercial feed; FF: pigs fed with fermented meal. Data are presented as mean ± SEM (*n* = 6).

We first characterized the influence of FF on the mRNA expression of Toll-like receptors (TLRs) and two antimicrobial peptide-encoding genes in the blood. The results are presented in Figure 2. The mRNA abundance of TLR3, TLR4, TLR6, and TLR8 in FF-fed pigs was significantly increased (*P* < 0.05) compared with that in the control group. Furthermore, the impact of FF on serum inflammatory factors was determined. Cytokines, as a series of small peptide molecules, are vital in the modulation of immunity and inflammatory responses (22). As shown in Figure 3, we found no statistical difference between the two groups. These results indicate that FF administration increased the mRNA expression of TLRs in the blood.

### Effect of FF on Composition of Intestinal Mucosal-Associated Microbiota

We used bacterial 16S rRNA sequencing based on V4–V5 hypervariable regions to analyze the impact of FF supplementation on duodenal mucosa- and colonic mucosa-associated microbiota. The mean ± SEM values of duodenal mucosa and colonic mucosa for each sample were 59,762 ± 4,560 and 51,272 ± 2,109, respectively. Using a pairwise identity threshold of 97% packing sequence, each sample showed an average operating taxon of 892 ± 119 (duodenal mucosa) and 909 ± 24 (colonic mucosa). Meanwhile, FF exhibited no statistical difference in terms of diversity of microbiota, as evidenced by similar richness estimators (ACE and Chao 1 index) and diversity indices (Shannon and Simpson indices), compared with control subjects (Table 1).

**Table 1:** Diversity estimation of the 16S.

**Items**	**Duodenal mucosa**			**Colonic mucosa**		
	**Ctrl**	**FF**	***P*-value**	**Ctrl**	**FF**	***P*-value**
Ace	932.58 ± 86.36	1,502.61 ± 207.95	0.142	1,066.42 ± 46.22	993.77 ± 32.24	0.226
Chao 1	927.22 ± 82.17	1,517.51 ± 204.76	0.148	1,063.58 ± 51.49	992.19 ± 37.17	0.287
Shannon	6.97 ± 0.15	4.84 ± 0.66	0.075	7.47 ± 0.19	7.25 ± 0.26	0.516
Simpson	0.96 ± 0.04	0.80 ± 0.06	0.128	0.98 ± 0.01	0.97 ± 0.02	0.455

*Ctrl, pigs fed with normal commercial feed; FF, pigs fed with fermented meal. Data are presented as the mean ± SEM (n = 6)*.

Next, we observed that Firmicutes and Bacteroidetes were the most predominant phyla in the duodenal and colonic mucosa of the control pigs at the phylum level (Tables 2, 3). The FF diet contained a lower proportion of Tenericutes (*P* < 0.05) in the duodenal mucosa-associated microbiota. Meanwhile, the FF diet had a higher proportion of Proteobacteria (*P* < 0.01) and a higher proportion of Acidobacteria (*P* < 0.05), but a lower proportion of Tenericutes in the colonic mucosa (*P* < 0.05).

**Table 2:** Relative abundance of microbial phylum (percentage) in the duodenal mucosa-associated microbiota of pigs in the FF and control groups.

**Phylum**	**Ctrl**	**FF**	***P*-value**
Firmicutes	59.15 ± 12.17	57.72 ± 8.16	0.939
Proteobacteria	1.03 ± 0.10	21.45 ± 9.43	0.175
Bacteroidetes	32.47 ± 9.96	7.91 ± 2.16	0.136
Spirochaetes	1.22 ± 0.34	0.34 ± 0.12	0.123
Unclassified phylum	4.19 ± 3.22	8.80 ± 6.66	0.665
Tenericutes	1.40 ± 0.28	0.45 ± 0.13	0.022
Actinobacteria	0.12 ± 0.03	2.23 ± 0.69	0.088
Chloroflexi	0.00 ± 0.01	0.72 ± 0.34	0.180
Cyanobacteria	0.24 ± 0.06	0.06 ± 0.02	0.116
Acidobacteria	0.02 ± 0.02	0.24 ± 0.12	0.238
Verrucomicrobia;	0.14 ± 0.08	0.08 ± 0.02	0.552

*Ctrl, pigs fed with normal commercial feed; FF, pigs fed with fermented meal. Data are presented as mean ± SEM (control n = 4, FF n = 6)*.

**Table 3:** Relative abundance of microbial phylum (percentage) in the colonic mucosa-associated microbiota of pigs in the FF and control groups.

**Phylum**	**Ctrl**	**FF**	***P*-value**
Firmicutes	47.98 ± 5.46	50.54 ± 3.83	0.709
Proteobacteria	1.43 ± 0.13	0.87 ± 0.06	0.007
Bacteroidetes	44.10 ± 5.53	42.25 ± 17.25	0.789
Spirochaetes	1.84 ± 0.37	3.26 ± 0.75	0.132
Unclassified phylum	0.47 ± 0.04	0.72 ± 0.39	0.354
Tenericutes	3.47 ± 0.55	1.66 ± 0.29	0.022
Actinobacteria	0.19 ± 0.03	0.34 ± 0.10	0.222
Planctomycetes	0.04 ± 0.03	0.18 ± 0.11	0.286
Verrucomicrobia	0.27 ± 0.08	0.06 ± 0.04	0.043
Cyanobacteria	0.21 ± 0.09	0.11 ± 0.07	0.349
Euryarchaeota	0.14 ± 0.07	0.12 ± 0.05	0.841

*Ctrl, pigs fed with normal commercial feed; FF, pigs fed with fermented meal. Data are presented as mean ± SEM (n = 6)*.

At the family level, we observed that BS11_gut_group and Rhodospirillaceae tended to decrease in terms of relative abundance after FF administration in the duodenal mucosa samples (0.05 < *P* < 0.10) (Table 4). At the same time, the results showed that fermented feeding significantly increased the proportion of Rikenellaceae and Christensenellaceae, whereas the abundance of Lachnospiraceae, Streptococcaceae, Veillonellaceae, and Succinivibrionaceae decreased in the colonic mucosa samples (*P* < 0.05) (Table 5). Collectively, the FF had a minimal effect on duodenal mucosa microbiota and shaped the colonic mucosa microbiota.

**Table 4:** Relative abundance (percentage) for the top 30 most abundant family in the duodenal mucosa-associated microbiota of pigs in the FF and control groups.

**Items**	**Ctrl**	**FF**	***P*-value**
Lactobacillaceae	24.85 ± 19.52	23.33 ± 12.33	0.959
Prevotellaceae	15.09 ± 4.58	2.77 ± 0.83	0.114
Clostridiaceae_1	9.85 ± 6.44	2.79 ± 0.96	0.439
Ruminococcaceae	8.29 ± 2.33	6.43 ± 2.27	0.656
Bacteroidales_S24-7_group	7.96 ± 2.49	2.08 ± 0.58	0.148
Lachnospiraceae	6.36 ± 1.78	2.90 ± 1.00	0.228
Unclassified family	6.19 ± 3.45	10.56 ± 6.90	0.665
Rikenellaceae	3.52 ± 1.24	0.79 ± 0.22	0.169
Veillonellaceae	2.30 ± 0.97	5.43 ± 1.68	0.247
Porphyromonadaceae	2.11 ± 0.77	0.87 ± 0.26	0.285
Bacteroidales_RF16_group	1.94 ± 0.54	0.53 ± 0.16	0.121
Streptococcaceae	1.76 ± 0.64	0.46 ± 0.27	0.202
Peptostreptococcaceae	1.70 ± 0.57	3.06 ± 1.73	0.577
Erysipelotrichaceae	1.32 ± 0.51	2.91 ± 1.63	0.495
Spirochaetaceae	1.22 ± 0.34	0.33 ± 0.12	0.123
Bacteroidaceae	0.90 ± 0.34	0.17 ± 0.05	0.182
Acidaminococcaceae	0.86 ± 0.24	0.35 ± 0.11	0.187
Christensenellaceae	0.78 ± 0.24	1.18 ± 0.45	0.562
Family_XIII	0.51 ± 0.19	0.51 ± 0.10	0.988
Anaeroplasmataceae	0.19 ± 0.08	0.01 ± 0.01	0.161
Mycoplasmataceae	0.18 ± 0.10	0.00 ± 0.00	0.229
Clostridiales_vadinBB60_group	0.17 ± 0.06	0.05 ± 0.02	0.181
P-2534-18B5_gut_group	0.17 ± 0.06	0.03 ± 0.01	0.164
Alcaligenaceae	0.16 ± 0.04	0.09 ± 0.03	0.351
BS11_gut_group	0.15 ± 0.05	0.01 ± 0.01	0.086
Peptococcaceae	0.13 ± 0.05	0.06 ± 0.02	0.395
Campylobacteraceae	0.12 ± 0.04	0.47 ± 0.29	0.398
Rhodospirillaceae	0.10 ± 0.03	0.02 ± 0.01	0.099
Helicobacteraceae	0.09 ± 0.02	0.22 ± 0.15	0.523
Succinivibrionaceae	0.09 ± 0.03	0.00 ± 0.00	0.131
Neisseriaceae	0.08 ± 0.03	0.05 ± 0.02	0.583

*Ctrl, pigs fed with normal commercial feed; FF, pigs fed with fermented meal. Data are presented as mean ± SEM (control n = 4, FF n = 6)*.

**Table 5:** Relative abundance (percentage) for the top 30 most abundant family in the colonic mucosa-associated microbiota of pigs in the FF and control groups.

**Items**	**Ctrl**	**FF**	***P*-value**
Prevotellaceae	25.76 ± 5.88	13.43 ± 2.67	0.098
Ruminococcaceae	17.80 ± 2.51	22.44 ± 2.01	0.180
Lachnospiraceae	11.61 ± 0.76	8.62 ± 0.72	0.017
Bacteroidales_S24-7_group	7.89 ± 1.50	10.15 ± 1.01	0.245
Streptococcaceae	5.22 ± 1.68	0.05 ± 0.02	0.027
Unclassified family	5.38 ± 0.75	4.61 ± 0.49	0.159
Rikenellaceae	4.53 ± 0.41	10.38 ± 1.61	0.014
Clostridiaceae_1	3.16 ± 0.97	1.40 ± 0.21	0.134
Bacteroidales_RF16_group	2.46 ± 0.56	2.29 ± 0.61	0.843
Veillonellaceae	2.15 ± 0.36	0.35 ± 0.18	0.003
Porphyromonadaceae	1.84 ± 0.35	3.75 ± 0.87	0.083
Spirochaetaceae	1.84 ± 0.37	3.26 ± 0.75	0.132
Peptostreptococcaceae	1.71 ± 0.40	1.70 ± 0.29	0.985
Acidaminococcaceae	1.47 ± 0.08	1.23 ± 0.29	0.446
Erysipelotrichaceae	1.41 ± 0.17	0.95 ± 0.12	0.057
Christensenellaceae	1.35 ± 0.40	3.43 ± 0.63	0.022
Family_XIII	0.66 ± 0.07	0.91 ± 0.13	0.141
Bacteroidaceae	0.60 ± 0.16	0.72 ± 0.13	0.566
Lactobacillaceae	0.60 ± 0.32	8.24 ± 5.47	0.221
Clostridiales_vadinBB60_group	0.42 ± 0.11	0.26 ± 0.03	0.221
Anaeroplasmataceae	0.36 ± 0.08	0.18 ± 0.06	0.109
P-2534-18B5_gut_group	0.27 ± 0.06	0.14 ± 0.03	0.118
Campylobacteraceae	0.26 ± 0.08	0.12 ± 0.03	0.140
Bacteroidales_BS11_gut_group	0.25 ± 0.13	0.65 ± 0.32	0.290
Succinivibrionaceae	0.22 ± 0.07	0.02 ± 0.02	0.037
Alcaligenaceae	0.19 ± 0.04	0.11 ± 0.05	0.276
Fibrobacteraceae	0.14 ± 0.05	0.04 ± 0.03	0.127
Peptococcaceae	0.13 ± 0.02	0.09 ± 0.01	0.123
Unidentified_thermoplasmatales	0.12 ± 0.07	0.04 ± 0.04	0.401
Enterobacteriaceae	0.11 ± 0.06	0.31 ± 0.10	0.125
Coriobacteriaceae	0.10 ± 0.02	0.12 ± 0.02	0.587

*Ctrl, pigs fed with normal commercial feed; FF, pigs fed with fermented meal. Data are presented as the mean ± SEM (n = 6)*.

### Effect of FF on sIgA Concentration in Intestinal Mucosa of Pigs

**Figure 4:**
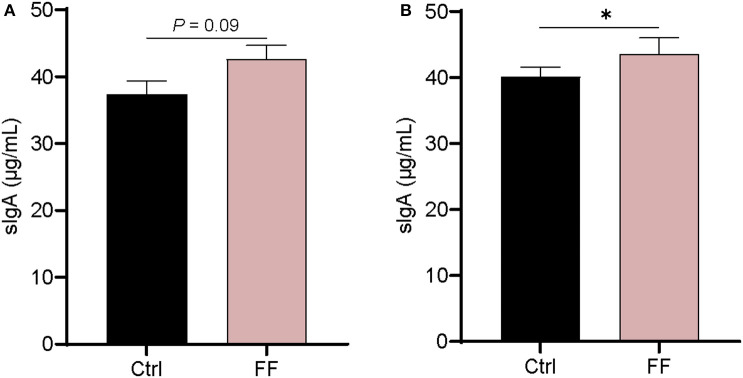
Effect of FF on the content of sIgA in the colonic mucosa. Ctrl, pigs fed with normal commercial feed; FF, pigs fed with fermented meal. Data are presented as mean ± SEM (*n* = 6). ^*^P < 0.05. **(A)** Duodenal mucosa; **(B)** Colonic mucosa.

IgA, as the most enriched immunoglobulin in mammals, is mainly secreted from mucous membranes (23). It is reported that mucosa-associated microbiota is critically important for intestinal production and secretion of sIgA (24). As shown in Figure 4, there was no significant difference in the concentration of sIgA in the duodenal mucosa. In the colon mucosa, the concentration of sIgA in FF-fed pigs was significantly increased compared with that in control animals (*P* < 0.05). Overall, the impact of FF on sIgA concentration in the intestinal mucosa of pigs mainly occurred in the colonic mucosa.

### Correlations Between Colonic Mucosa-Associated Microbiota and Immunity-Related Genes and Colonic sIgA

**Figure 5:**
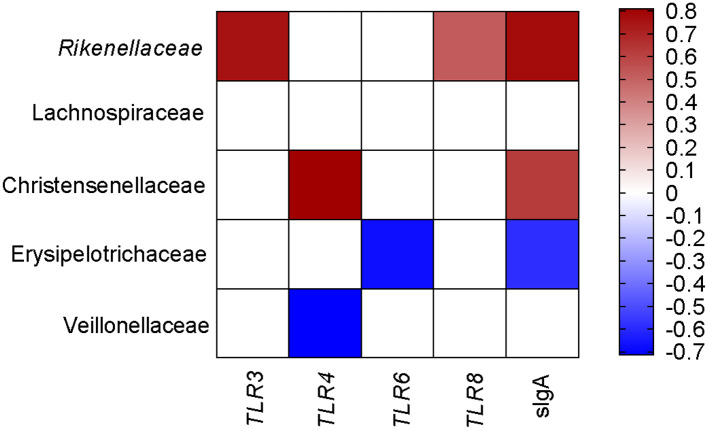
Correlation analysis between colonic microbial composition and genes related to blood immunity and sIgA, which were significantly influenced by the FF diet in the colonic of pigs compared to the Ctrl diet. Ctrl, pigs fed with normal commercial feed; FF, pigs fed with fermented meal. The color represents a significant correlation (*P* < 0.05), and the intensity of the colors represents the degree of association. Red represents significantly positive correlation and blue represents significantly negative correlation (*n* = 6).

To comprehensively analyze the relationship between pig blood immune-related genes, sIgA, and colonic mucosa-associated microbiota, the Spearman correlation coefficient was calculated to generate the correlation matrix. As shown in Figure 5, we observed that TLR3, TLR8, and sIgA were positively associated with the proportion of Rikenellaceae. The gene expression of TLR4 was positively associated with the proportion of Christensenellaceae but negatively related to the proportion of Veillonellaceae. sIgA was positively associated with the proportion of Christensenellaceae but negatively related to the proportion of Erysipelotrichaceae. Meanwhile, the gene expression of TLR6 was negatively associated with the proportion of Erysipelotrichaceae. Taken together, these findings showed that colonic mucosa microbiota was associated with the expression of immunity-related genes and colonic sIgA.

## Discussion

Over the past several decades, antibiotic resistance and antibiotic residues have become a growing problem worldwide (25, 26). FF, a widely investigated diet that has been used as an antibiotic growth promoter (27), was recently shown to drive down feed prices through the use of industrial by-products in livestock (28, 29). In this study, we compared the effects of FF and unfermented corn–soybean diet on blood immunity, serum inflammatory factor, and intestinal (duodenum and colon) mucosa-associated microbiota composition in grower–finisher pigs. Our data indicated that fermented feeding modulated the expression of the blood gene and selectively modified the mucosa-associated microbiota composition.

Corn and soybean meal account for about 70% of global feed consumption. However, the utility of conventional corn and soybean meal mixed is limited by several antinutrient factors that inhibit nutrient bioavailability and impair animal health (30). Solid-state fermentation has provided a solution, which has been widely used in China, to improve the nutritional quality of feed (31). In the present study, we used a complete fermented corn–soybean meal (100% fermented), which may increase manufacturing costs in conventional swine production. Hence, further studies specifically focusing on different inclusion levels of FF are warranted to explore the appropriate dose of supplementary feeds to help increase income in swine production.

It has been reported that TLRs play a vital role in immunity (32). One previous study showed that the expression of TLRs may reflect the status of disease resistance of pigs (33, 34). In our study, we found that the mRNA abundance of TLR3, TLR4, TLR6, and TLR8 in FF-fed pigs was significantly higher (*P* < 0.05), indicating that the FF diet could influence the gene expression of TLRs. Interestingly, we found no significant difference between the two antimicrobial peptides (PBD-1 and PR-39), whereas the mRNA abundances of PBD-1 and PR39 in the duodenum were significantly increased (*P* < 0.05) in our previous study (19). In the same study, we had observed high concentrations of immunoglobulins in the serum samples, and intestinal inflammation was not detected, indicating that the gene expression of both groups was in normal ranges. Furthermore, it is essential to develop an *in vivo* pathogenic challenge model as a further study to confirm that the FF diet enhances immune performance.

There are extensive studies on the beneficial effects of FF on pig gut microbiota, but so far only a few studies have focused on mucosa-associated microbiota. Nevertheless, researchers had found that colonic mucosa-associated microbiota plays an important role in host metabolism and immune homeostasis (35, 36). In the present study, we found that FF had no significant effect on the number of OTUs, whereas the abundance of certain phyla was significantly affected. The proportion of Proteobacteria and Acidobacteria were significantly increased (*P* < 0.05), whereas the proportion of Tenericutes was decreased in the colonic mucosa (*P* < 0.05) in response to FF meal. We further analyzed the influence of the FF diet on colonic mucosa-associated microbiota at the family level. It has been reported that the increased proportion of Christensenellaceae (a butyrate producer) was beneficial for the sow's health (37), and our data found that FF diet significantly increased the proportion of Christensenellaceae. In addition, a previous study found that the enhanced ratio of Christensenellaceae might promote a lean host phenotype in pigs (38). Meanwhile, the family of Rikenellaceae may be related to anaerobic metabolism (39). One study has shown that Erysipelotrichaceae was related to the digestion of protein and energy in dogs (40). In the present study, the significantly decreased proportion of Erysipelotrichaceae may provide benefit to the pig's gut health. In addition, some species of Veillonellaceae, as an opportunistic pathogen for animals, could be responsible for polymicrobial infections (41). Interestingly, in the present study, the proportion of Lachnospiraceae was significantly decreased in FF-fed pigs compared with that in the control group. In short, the FF diet shaped the colonic mucosa-associated microbiota.

Then we determined the concentration of sIgA in the duodenal mucosa and colonic mucosa. It has been reported that sIgA is the most abundant colonic antibody antigen known as “immune exclusion” (42). The result was consistent with previous studies (43, 44), which reported that diet could promote intestinal sIgA secretion through intestinal microbiota and could be beneficial to the animal's health (45). Furthermore, we analyzed the correlation between colonic mucosa-associated microbiota and sIgA, and the results indicated that sIgA was influenced by the intestinal mucosa-associated microbiota. Our data showed that FF is beneficial to swine gut health. Moreover, the tight junction in the gut should be taken into consideration in further studies as well as in potential practical applications.

FF meal altered the blood immunity and composition of colonic mucosa-associated microbiota in pigs. The colonic mucosa-associated microbiota was closely associated with the expression of blood immunity-related genes. For instance, Christensenellaceae, a bacterial family known to be abundant in the intestinal ecosystem, was positively correlated with lipid metabolism (46). Furthermore, our data showed that Christensenellaceae was also positively correlated with TLR4 expression in the blood and sIgA in the colonic mucosa. These lines of evidence suggest that the beneficial effect of FF might be attributable to the higher relative abundances of some beneficial bacteria on the colonic health of grower–finisher pigs. Further studies are necessary to confirm the roles of these gut bacteria in regulating swine health.

## Conclusion

Taken together, the findings of this study revealed that feeding complete fermented corn–soybean meal (100% fermented) to grower–finisher pigs could increase the expression of genes related to immunity in the blood, and changed the concentration of sIgA in the colonic mucosa and modified the microbiota community in the colonic mucosa. These findings highlight the positive role of FF diet in pig health. Furthermore, it would be interesting to explore the best dose for feed supplements, which we would like to focus on in our next study.

## Data Availability Statement

The datasets generated for this study can be found in the NCBI Repository [accession number: PRJNA607631].

## Ethics Statement

Animal protocols for this study were approved by the Animal Ethics Committee of Northwest A&F University (Yangling, Shaanxi, China).

## Author Contributions

XS and JL designed the study. JL, HC, XuZ, XiZ, XL, and JH helped performed the experiments and took samples. MZ and ZW analyzed the data. JL and MZ wrote and revised the manuscript. MZ, XL, JH, GY, and XS modified the manuscript. All authors reviewed the final manuscript.

## Conflict of Interest

The authors declare that the research was conducted in the absence of any commercial or financial relationships that could be construed as a potential conflict of interest.
