# Supplementary material for: Impact of Fermented Corn–Soybean Meal on Gene Expression of Immunity in the Blood, Level of Secretory Immunoglobulin A, and Mucosa-Associated Bacterial Community in the Intestine of Grower–Finisher Pigs
Source: Front Vet Sci. 2020 Jun 2;7:246. doi: 10.3389/fvets.2020.00246 (PMC7325983; doi:10.3389/fvets.2020.00246)
Supplement: Supplementary file 1 [file Data_Sheet_1.docx]

Supplementary materials for:

Impact of Fermented Corn-Soybean Meal on Gene Expression of Immunity in the Blood, Level of Secretory Immunoglobulin A, and Mucosa-Associated Bacterial Community in the Intestine of Grower–Finisher Pigs

**Supplementary Table 1.** Ingredients and nutrient component of experimental meals.

| **Ingredients** | **Ratio/%** | | **Nutrient component^2^** | **Content/%** | |
| --- | --- | --- | --- | --- | --- |
|  | **Ctrl** | **FF** |  | **Ctrl** | **FF** |
| Corn | 67.0 | 67.0 | Dry matter | 89.42 | 76.03 |
| Soybean meal | 21.0 | 21.0 | Gross energy | 12.78 | 12.65 |
| Wheat bran | 8.0 | 8;0 | Crude protein | 15.21 | 16.36 |
| Premix^1^ | 4.0 | 4.0 | Crude fiber | 2.48 | 2.96 |
| Total | 100 | 100 | Crude fat | 2.02 | 1.40 |
|  |  |  | Crude ash | 5.95 | 5.21 |
|  |  |  | Acid Detergent Fiber | 4.04 | 4.02 |
|  |  |  | Neutral detergent fiber | 13.24 | 11.78 |

^1^Premix supplied per kilogram of meal: VD_3_, 2800 IU; VE, 26 mg; VK, 2 mg; VB1, 50 mg; VB6, 3 mg; VA, 6480 IU; VB2, 4 mg; VB12, 0.03 mg; pantothenic acid, 9 mg; nicotinic acid, 20 mg; choline chloride, 300 mg; biotin, 0.2 mg; Fe, 200 mg; Cu, 95 mg; Mn, 30 mg; folic acid, 1.2 mg; Zn 100 mg; I, 0.35 mg; Se, 0.36 mg; P 0.1%; NaCl, 0.5%; lysine, 0.1%; Ca, 0.9 %.^2^Nutrient contents are calculated values. Ctrl: pigs fed with normal commercial feed; FF: pigs fed with fermented meal.

**Supplementary Table 2.** Primers sequences for qRT-PCR

| **Genes** | **Primer sequence (5′-3′)** | **Product (bp)** | **GenBank accession** |
| --- | --- | --- | --- |
| *TLR1* | F:TTAGGAGACTCTTACGGGGAA  R:ATTTACTGCGGTGCTGACTGA | 135 | NM_001031775.1 |
| *TLR2* | F:GTTTTACGGAAATTGTGAAACTG  R:TCCACATTACCGAGGGATTT | 128 | NM_213761.1 |
| *TLR3* | F:GCATTGCCTGGTTTGTTAGTTG  R:TGTATCAAAAAGAATCACTGGGAG | 122 | NM_001097444.1 |
| *TLR4* | F:ATATGGCAGAGGTGAAAGCAC  R:GAAGGCAGAGATGAAAAGGGG | 125 | NM_001113039.2 |
| *TLR5* | F:AGTTCCGGGGATTTTGTTTCA  R:GCATAAGTAGGCATCGTATTTGTAT | 110 | [NM_001348771.1](https://www.ncbi.nlm.nih.gov/entrez/viewer.fcgi?db=nucleotide&id=1147710170) |
| *TLR6* | F:CATCACCAGCCTCAAGCATTT  R:TTCAGTTGTGTCAAGTTGCCAA | 90 | NM_213760.2 |
| *TLR7* | F:ATAGCGAGCATCACTCCAGCC  R:TAATCTGCTGCCTTCTGGTGC | 127 | NM_001097434.1 |
| *TLR8* | F:CTGGGATGCTTGGTTCATCT  R:CATGAGGTTGTCGATGATGG | 150 | NM_214187.1 |
| *TLR9* | F:ACAATGACATCCATAGCCGAGT  R:CAGATCGTTGCCGCTAAAGT | 80 | NM_213958.1 |
| *PBD-1* | F:TGCCACAGGTGCCGATCT  R:CTGTTAGCTGCTTAAGGAATAAAGGC | 105 | NM_213838.1 |
| *PR39* | F:CCACTCCATCACCGTTTTCC  R:CAAGGCCACCTCCGTTTT | 129 | NM_214450.1 |
| *GAPDH* | F:AGGTCGGAGTGAACGGATTTG  R:ACCATGTAGTGGAGGTCAATGAAG | 118 | NM_001206359.1 |

**Supplementary Table 3.** Primers used for encoding the V4–V5 region of 16S rRNA

| **Direction** | **Primer** |
| --- | --- |
| Forward | 5′-AATGATACGGCGACCACCGAGATCTACAC-i5[^a^](https://www.ncbi.nlm.nih.gov/pmc/articles/PMC5330390/table/T2/#t2n1)TATGGTAATTGTGTGCCAGCMGCCGCGGTAA-3′ |
| Reverse | 5′-CAAGCAGAAGACGGCATACGAGAT-i7[^a^](https://www.ncbi.nlm.nih.gov/pmc/articles/PMC5330390/table/T2/#t2n1)-AGTCAGTCAGGCCCCGTCAATTCMTTTRAGT-3′ |
